# Progress and innovations of gene cloning in wheat and its close relatives

**DOI:** 10.1007/s00122-025-04897-w

**Published:** 2025-04-29

**Authors:** Zuzana Korchanová, Alexander Milovanov, Miroslav Švec, Jaroslav Doležel, Jan Bartoš, Miroslav Valárik

**Affiliations:** 1 Centre of Plant Structural and Functional Genomics, Institute of Experimental Botany of the Czech Academy of Sciences, 77900 Olomouc, Czech Republic; 2 Department of Cell Biology and Genetics, Faculty of Science, Palacký University, 77900 Olomouc, Czech Republic; 3 Department of Botany, Faculty of Natural Sciences, Comenius University in Bratislava, Bratislava, 84104 Slovakia

## Abstract

**Key message:**

Wheat and its close relatives have large and complex genomes, making gene cloning difficult. Nevertheless, developments in genomics over the past decade have made it more feasible.

**Abstract:**

The large and complex genomes of cereals, especially bread wheat, have always been a challenge for gene mapping and cloning. Nevertheless, recent advances in genomics have led to significant progress in this field. Currently, high-quality reference sequences are available for major wheat species and their relatives. New high-throughput genotyping platforms and next-generation sequencing technologies combined with genome complexity reduction techniques and mutagenesis have opened new avenues for gene cloning. In this review, we provide a comprehensive overview of the genes cloned in wheat so far and discuss the strategies used for cloning these genes. We highlight the advantages and drawbacks of individual approaches and show how particular genomic progress contributed to wheat gene cloning. A wide range of new resources and approaches have led to a significant increase in the number of successful cloning projects over the past decade, demonstrating that it is now feasible to perform rapid gene cloning of agronomically important genes, even in a genome as large and complex as that of wheat.

**Supplementary Information:**

The online version contains supplementary material available at 10.1007/s00122-025-04897-w.

## Introduction

Wheat (*Triticum*) was one of the first domesticated food crops, and nowadays, it dominates the world’s production in habitable areas from 67°N in Scandinavia to 45°S in Argentina, including elevated regions in the tropics and subtropics (Feldman 1995). Its importance as a staple food is challenged only by rice and maize (www.fao.org). The domestication of wheat dates back approximately 10,000 years ago. At that time, the first farmers began to intentionally select traits that favored mutant types over their wild counterparts, without any knowledge of the genetic background. Over time, this human-guided selection resulted in varieties that were more suitable for agriculture. As a result, wheat has spread all over the world and become a staple food for 35% of the world’s population, providing on average about 20% of calories (in some regions 50%) and protein in the global human diet (Shiferaw et al. 2013). Consequently, stable yields as well as the need to increase them to feed ~ 9.7 billion humans in 2050 have become crucial to ensuring food security. The first major increase in wheat yields occurred in the 1960 s during the era called the “Green Revolution.” At that time, the introduction of the dwarfing genes *Rht-B1b* and *Rht-D1b* helped to increase wheat yields by 5–10% (Gale et al. 1985) and prevent the famine that was threatening the developing countries. Implementation of new genes/alleles is indispensable for effective breeding to ensure food security for the fast-growing human population. Since the advent of molecular markers, genetic mapping studies have identified a wealth of loci for various traits in wheat or its wild relatives. The results of these studies are usually markers tightly linked to the gene of interest. Although tightly linked markers have a high potential to ensure the transfer and maintenance of desired genes/loci in breeding germplasms by marker-assisted selection (MAS), the “perfect marker,” which never segregates from the desired trait/gene, can only be designed from the gene/genetic feature determining the trait variability. Knowledge of the gene sequence is, therefore, a key factor for its effective use in molecular breeding as well as for its functional analysis.

The process of identifying a gene sequence that is responsible for the variability of a particular trait is known as “gene cloning.” The process can be divided into two parts—candidate gene(s) identification and functional validation. Candidate gene identification is the initial part of the gene cloning process, where potential genes responsible for the observed trait variability are pinpointed. Once candidate gene(s) are identified, they are subjected to functional validation to confirm their involvement in the trait phenotype (reviewed by Adamski et al. 2020).

In the present review, we aim to provide a comprehensive overview of the approaches and methods employed in candidate gene(s) identification, with a focus on the large and complex wheat genome. We show how advances in genomics and next-generation sequencing (NGS) technologies have played a crucial role in facilitating this process. Additionally, we discuss the advantages and disadvantages of each cloning method, which we believe may help researchers to choose an appropriate cloning method.

## Historical insights into wheat gene cloning

The first approaches used in wheat gene cloning were homology-based and map-based cloning. Homology-based cloning identifies genes based on homology to previously cloned and characterized genes in other species and is limited to genes conserved between the species. In contrast, map-based (or positional) cloning allows the cloning of almost any gene of interest without any assumption of a gene sequence. Therefore, map-based cloning was the method of choice for gene identification until novel approaches began to emerge. 

**Fig. 1:**
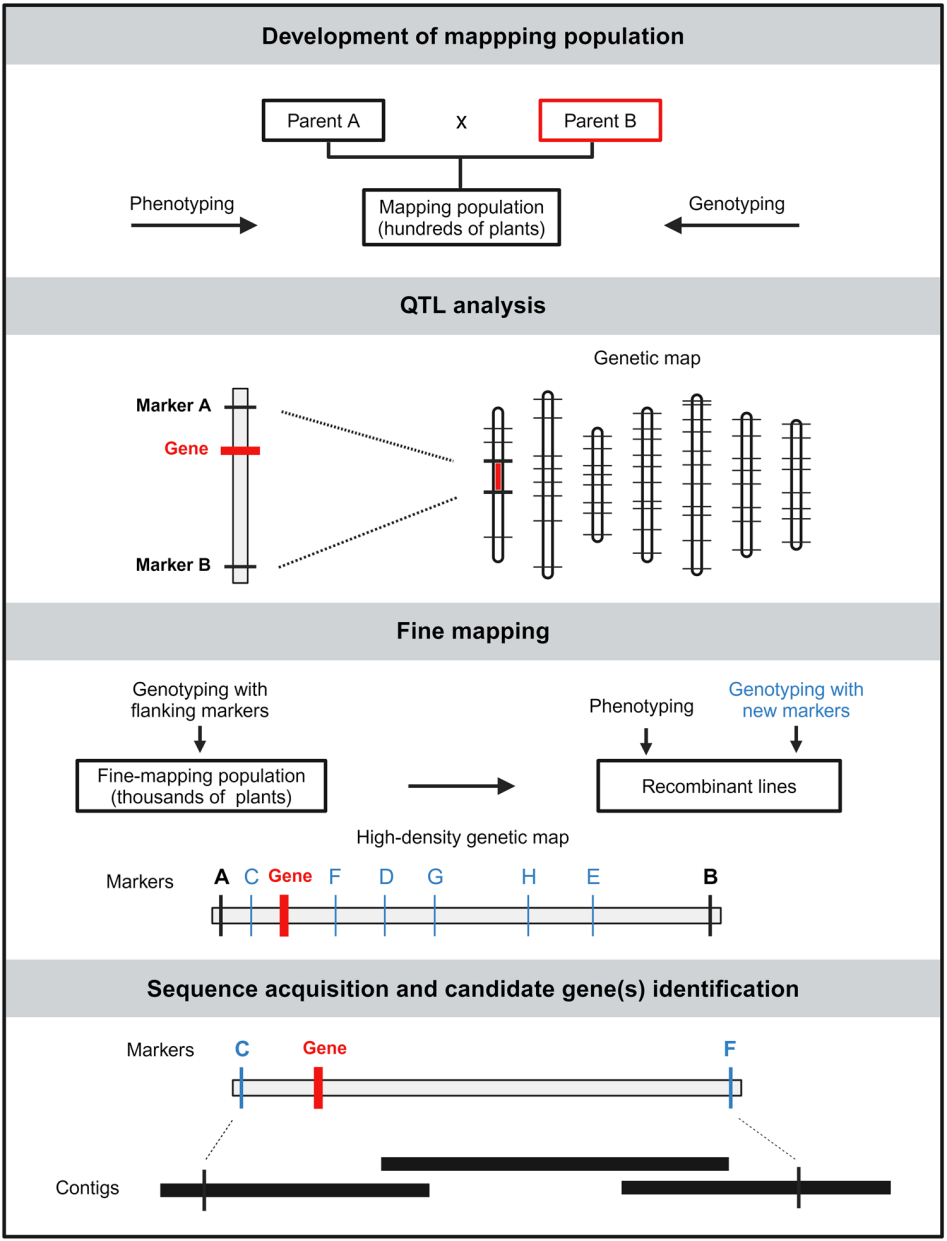
Workflow of map-based cloning. The first step of map-based cloning involves the development of a mapping population by crossing parents with contrasting phenotypes. The mapping population is genotyped with markers equally distributed across the genome and phenotyped for the trait of interest. Obtained markers are used for the construction of genetic map, which is used together with the phenotypes for QTL analysis. QTL analysis results in the identification of locus/loci determining the trait of interest. In the next step, recombinant lines, obtained by genotyping individuals descendent from plants heterozygous in the mapped region with flanking markers (Marker A and B), are phenotyped and genotyped with new markers (C, D, E, F, G, H) to saturate the mapped locus. This results in markers flanking the target gene within a more narrowly defined interval. The final step is to obtain the sequence between the markers flanking the gene of interest. Created with BioRender

Map-based cloning relies on genetic and QTL mapping and multiple molecular approaches that result in a stepwise localization of the gene within the genome and subsequently in its sequence identification (Fig. 1). The first step is the development of mapping population by the cross of parents contrasting in phenotype. Usually, an F_2_, DH, or RIL mapping population counting 100–200 individuals is enough for rough genetic mapping. Segregating mapping population is genotyped with molecular markers equally distributed across the genome and phenotyped for the trait of interest. A genetic map containing hundreds to thousands of markers is constructed using software packages for genetic map construction (Lander et al. 1987; Stam 1993; Ronin et al. 2017; Heffelfinger et al. 2017). The genetic map and the phenotypic data collected for all progenies of the mapping population are used for quantitative trait locus (QTL) analysis. The QTL analysis determines how many loci are involved in the variability of the trait. If more than one locus is involved, it is necessary to establish the genetic background to the stage where only the locus selected for cloning contributes to phenotypic variability—a process referred to as “mendelization” (e.g., Athiyannan et al. 2022; Korchanová et al. 2022). The identified locus/loci are often delimited to a size of several centimorgans (cM), which may represent tens or hundreds of candidate genes. Therefore, further fine-mapping is required to identify the causal gene. The aim of fine-mapping is to increase the resolution of the genetic map at the position where the gene was identified. Usually, thousands of individuals descendent from plants heterozygous in the mapped region are genotyped with flanking markers. Individuals showing recombination between flanking markers are selected for further analysis, which involves phenotyping and saturating the region with new markers. The target region can be delimited to different extents depending on factors such as population size, marker density, and recombination frequency. A good example is the saturation of *QPm.GZ1-7 A* (Korchanová et al. 2022), where the powdery mildew resistance locus was narrowed from the original 46.9 Mb to 1.18 Mb using lines from F_2_ to F_5_ generations. Once the target locus is fine-mapped and markers flanking the gene of interest are identified within a reasonably small interval (< 2 cM, Keller et al. 2005), a sequence spanning the target region is generated and annotated in order to identify candidate gene(s).

The first markers used for genetic map construction were based on Restriction Fragment Length Polymorphism (RFLP). In 1989, the International Triticeae Mapping Initiative (https://wheat.pw.usda.gov/ITMI/index.html) was established to develop and share RFLP genetic maps for the genomes of *Triticeae*. However, RFLP markers alone were insufficient to create high-density genetic maps, and it became obvious that additional markers were required to enhance the density of genetic maps. Consortia were built internationally to develop and map other markers like SSR (Simple Sequence Repeats) or EST (Express Sequence Tag). For example, the International Triticeae EST Cooperative (ITEC) was launched in 1998 to produce EST markers, given that there were only 6 wheat ESTs and a handful of other *Triticeae* ESTs available at that time. By 2013, the 1,329,396 ESTs from *Triticum* species became publicly available (https://wheat.pw.usda.gov/genome/). Emerged databases that provided access to all existing RFLPs, SSRs, ESTs, and other markers highly supported genetic mapping in wheat, for which reference genome sequence was not available (e.g., GrainGenes—wheat.pw.usda.gov/GG3/; KOMUGI—shigen.nig.ac.jp/wheat/komugi). In addition to markers developed from *Triticum* species, researchers could derive additional markers by conducting collinearity studies with closely related species, for which the reference genome sequence was available (*Brachypodium distachyon*, rice, and sorghum). This was particularly exploited in the fine-mapping stage of map-based cloning. Although the genomes of wheat relatives were successfully used in several projects (Liu et al. 2013; Griffiths et al. 2006; Yan et al. 2003), they represented only an approximate model of the wheat genome because of the frequent microcollinearity exceptions (Valárik et al. 2006; Delseny 2004; Distelfeld et al. 2004). The major shift in marker development occurred with the release of the draft bread wheat genome sequence by the International Wheat Genome Sequencing Consortium (IWGSC) in 2014 (IWGSC 2014).

To obtain sequence information of the fine-mapped region, a physical map of the region had to be constructed. The physical map was constructed using a chromosome-walking approach based on screening libraries of large DNA fragments (50–200 kbp) cloned in bacterial artificial chromosomes (BACs). The flanking markers identified by genetic mapping were used to PCR screened 3D pools of the BAC library (Barillot et al. 1995; Nilmalgoda et al. 2003; Paux et al. 2008). The identified BAC clones were fingerprinted, assembled into a contig, and the BAC clones the most protruding toward the gene region were either BAC end or fully sequenced. In the next step, the acquired sequences were a source of new markers for the next step of the library screening. The process was repeated until the gene region between the flanking markers was covered by the Minimal Tilling Path (MTP—the BAC clones, which, with minimal overlap, represent the whole region). The MTP clones were fully sequenced, and the sequence of the locus region was assembled. The sequence was de novo annotated using various gene prediction software such as TriAnnot (Leroy et al. 2012) or Fgenesh +  + (Solovyev et al. 2006). The predicted candidate genes were validated by comparison with databases of ESTs, proteins, and reference genome sequences of the related species. Development of BAC libraries was for a long time limited to small genomes, and it had remained very difficult in large (> 5,000 Mb) and complex genomes with high content of repetitive elements (> 80%) such as those of cereals and especially wheat. For this reason, the first BAC libraries were constructed from two related diploid wheat species—*Triticum monococcum* (Lijavetzky et al. 1999) and *Aegilops tauschii* (Moullet et al. 1999). The *T. monococcum* library was created to clone *VRN1* (Yan et al. 2003) and *VRN2* (Yan et al. 2004) genes controlling vernalization response in *T. monococcum*. In addition, this library was later successfully used in map-based cloning of fungal disease resistance genes *Sr35* (Saintenac et al. 2013) and *Sr21* (Chen et al. 2018) as well as in sub-genome map-based cloning of *Q* (Faris et al. 2003), *Lr10* (Feuillet et al. 2003), or *Pm3b* (Yahiaoui et al. 2004) genes from hexaploid wheat. The first BAC library from tetraploid wheat (*T. turgidum* subsp. *durum*) was constructed (Cenci et al. 2003) to support map-based cloning of the *Gpc-B1* gene regulating senescence and improving grain protein, zinc, and iron content (Uauy et al. 2006). The later improvements in the quality and efficiency of BAC cloning also allowed the construction of BAC libraries for the whole hexaploid wheat genome comprising 1.2 million clones representing the 9.3 × coverage of the genome (Allouis et al. 2003; Nilmalgoda et al. 2003). However, it became obvious that agronomically important traits are frequently controlled by rare, genotype-specific alleles absent from the reference lines (the ones with the BAC libraries). It was therefore desirable to use a genomic library of the cultivar or line with the gene of interest. A few cultivar-specific BAC libraries were constructed to enable gene cloning (Rawat et al. 2016; Klymiuk et al. 2018; Li et al. 2020; Chen et al. 2020), including those from hexaploid wheat (Ni et al. 2017). However, the preparation and screening of libraries composed of more than a million BAC clones of polyploid wheat represented a laborious and tedious task. In 2000, Vrána et al. (2000) optimized a chromosomal flow sorting for wheat, which enabled dissection of the wheat genome into individual chromosomes or chromosome arms. BAC libraries constructed from flow-sorted chromosomes represented a sophisticated way how to facilitate the construction of physical maps as they (i) represented only a few percent of the genome; (ii) contained small number of clones; (iii) enabled to obtain high genome coverage; (iiii) did not contain homoeologous chromosomes in case of higher ploidy (Šimková et al. 2011). 

The emergence of databases offering comprehensive access to molecular markers along with the release of the draft bread wheat genome sequence and the progress in BAC library construction and screening significantly facilitated gene cloning in wheat. However, the recent advancements in sequencing technologies and bioinformatics tools have elevated the process to an entirely new level.

## Map-based cloning in the NGS era

The effort required for map-based gene isolation in wheat has dropped dramatically in recent years. The driving force is undoubtedly the advancement of sequencing technologies, which have simultaneously accelerated several steps of the process. The genotyping of mapping population has been significantly accelerated by the use of capture arrays or genotyping-by-sequencing (GBS) protocols (Miller et al. 2007; Elshire et al. 2011; Kilian et al. 2012) providing hundreds to thousands of molecular markers, usually based on single nucleotide polymorphism (SNP), in a single analysis. This enables construction of genetic maps with higher marker density, supporting more accurate QTL mapping. The designing of new markers for fine-mapping is facilitated by the availability of reference genome sequences or by the possibility of sequencing the respective chromosomes of parental lines (e.g., Janáková et al. 2019; Korchanová et al. 2022). The candidate gene identification can be achieved by anchoring the locus to the multiple reference genome sequences, which provide comprehensive insight into gene content. To date, high-quality reference genome sequences have been published for several hexaploid wheats—*T. aestivum* (Walkowiak et al. 2020; Sato et al. 2021; Zhu et al. 2021; Akpinar et al. 2022; Athiyannan et al. 2022; Aury et al. 2022; Kale et al. 2022; Jiao et al. 2025), *T. timopheevii* (Grewal et al. 2024), and for its relatives—*T. urartu* (Ling et al. 2018), *T. turgidum* ssp. *durum* (Maccaferri et al. 2019), *T. turgidum* ssp. *dicoccoides* (Zhu et al. 2019), *Ae. tauschii* (Wang et al. 2021; Zhou et al. 2021), *Ae. longisimma*, *Ae. spletoides, Ae. sharonensis* (Avni et al. 2022), *Ae. umbellulata* (Abrouk et al. 2023), *T. monococcum* (Ahmed et al. 2023) and can be accessed at GrainGenes (https://wheat.pw.usda.gov/GG3/). Nonetheless, if the gene of interest is absent from reference genome sequences, the sequence information from a line carrying the gene of interest must be generated. The tedious BAC library construction and chromosome walking can now be overcome by recently developed long-read sequencing platforms such as Pacific Bioscience (PacBio, https://www.pacb.com/) and Oxford Nanopore Technology (ONT, https://nanoporetech.com/). Athiyannan et al. (2022) generated de novo genome assembly by a combination of PacBio CCS (Hifi) sequencing, optical mapping, and Hi-C contact map to facilitate gene cloning of the stripe rust resistance loci *QYr.sgi-2B.1* (*Yr27*) and *QYr.sgi-4 A.1* identified in the South African bread wheat cultivar Kariega. Using only the PacBio CSS, they generated an assembly of 14.66 Gb (5,055 contigs) in length with a contig N50 length of 30.22 Mb, which is itself highly sufficient for obtaining the sequence of the mapped region. Another who successfully employed long-read sequencing, specifically ONT, in order to obtain a sequence of the mapped region was Li et al. (2023b). Long-read sequencing enables generation of megabase-sized scaffolds spanning the target regions within a few months, providing a highly efficient alternative to chromosome walking.

## Mutagenesis—a gateway to rapid gene cloning

**Fig. 2:**
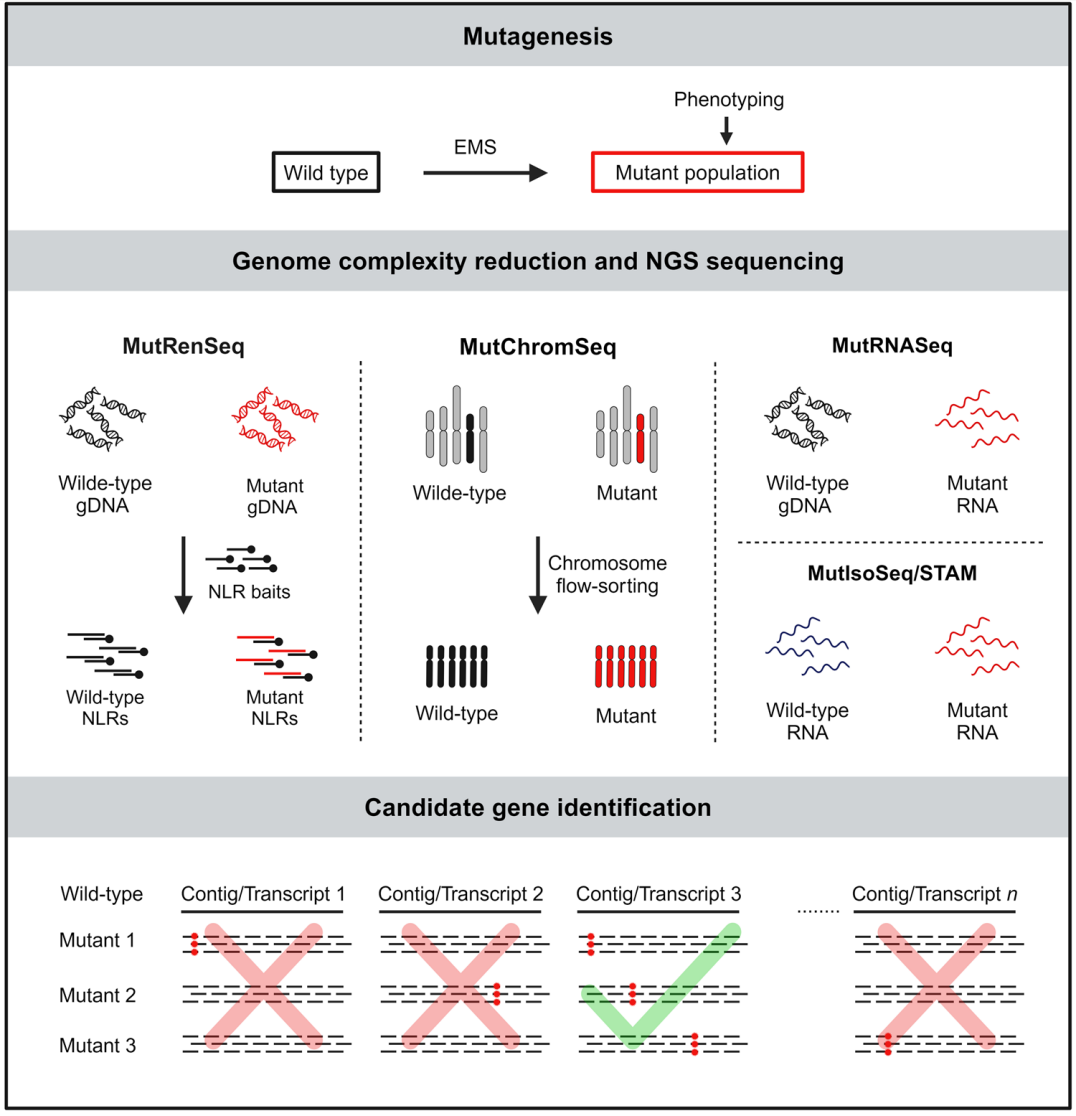
Workflow of mutagenesis-based cloning approaches. The first step of mutagenesis-based approaches involves development of mutant population and identification of loss-of-function mutants. Depending on the method used, the next step is genome complexity reduction. In MutRenSeq, wild-type parent and several independent EMS-derived mutants are subjected to disease resistance gene enrichment targeting the NLR sequences. The NLR sequences are NGS sequenced and wild-type reads are de novo assembled into NLR contigs. In MutChromSeq, the chromosome with the locus of interest from the wild-type parent and several independent EMS-derived mutants (black and red, respectively) are flow sorted. The chromosomes are NGS sequenced and wild-type reads are de novo assembled into contigs. In MutRNAseq and MutIsoSeq/STAM the complexity reduction is achieved by short-read transcriptome sequencing. The difference between these methods lies in sequencing of the wild type. In the case of MutRNAseq, the wild-type parent is subjected to long-read whole-genome sequencing, while in MutIsoSeq/STAM the wild-type parent is subjected to Iso-seq. The final step in all these approaches is the identification of candidate genes by mapping the reads from mutants to obtained contigs/transcripts of the wild type. The contig/transcript, for which a mutation is found in all examined mutants (Contig/Transcript 3), is subjected to candidate gene identification. Created with BioRender

Mutagenesis provides an efficient strategy to directly pinpoint a candidate gene. Cloning approaches utilizing mutagenesis (Fig. 2) are based on sequence comparison of multiple mutants to the wild-type genome. In general, they start by identifying loss-of-function mutants in mutated population. Several independent loss-of-function mutants together with the respective wild type are subjected to sequencing. Sequence reads from the independent mutants are mapped to the wild-type de novo assembly and analyzed for the single nucleotide variants between the wild-type contigs and mutant reads. Contig, for which a mutation is identified in all examined mutants, is subjected to the identification of candidate gene. The most widely used mutagen is ethyl-methanesulfonate (EMS), which produces random and relatively evenly distributed G/C to A/T transitions (Farrell et al. 2014; Shirasawa et al. 2016; Krasileva et al. 2017). The number of induced mutations after EMS treatment in diploids is usually around 5–11 SNPs per Mb on average (Henry et al. 2014; Jiao et al. 2016), while in tetraploids and hexaploids it is around 20–42 SNPs per Mb (Slade et al. 2005; Uauy et al. 2009; Henry et al. 2014; Krasileva et al. 2017), which makes the probability of obtaining a functional mutation in a target gene relatively high. The critical step in mutagenesis-based approaches is assessment of mutations. The advancement in sequencing technologies enabled the screening of induced mutations at the genome-wide level through whole-genome sequencing (Abe et al. 2012; Fekih et al. 2013). Unfortunately, the whole-genome re-sequencing of loss-of-function mutant lines is not an effective strategy for large and complex genomes such as wheat. Therefore, some form of genome complexity reduction is highly desirable.

One of the strategies for complexity reduction is through capture arrays (Krasileva et al. 2017). This was particularly applied in the cloning of disease resistance genes. Most disease resistance genes cloned in plants so far encode intracellular immune receptors of the NLR family (reviewed in Kourelis and van der Hoorn 2018). Mapping and cloning these genes have been the subject of several research and breeding programs. The first, who used conservative NLR sequences as an advantage to study disease resistance genes, was Jupe et al. (2013). They developed a capture array (bait library) that specifically enriches for the NLR coding sequences. The capture array comprises biotinylated oligonucleotide “baits,” whose sequences are based on sequence information of already identified NLR genes. The target DNA hybridizes with the baits and is used for NGS sequencing. Jupe et al. (2013) demonstrated that approximately 80% identity is sufficient for NLR genes enrichment. Steuernagel et al. (2016) utilized RenSeq (resistance gene enrichment sequencing) in combination with EMS mutagenesis, named MutRenSeq (Fig. 2), to clone the stem rust resistance genes *Sr22* and *Sr45* from common wheat. They designed a cereal NLR bait library containing 60,000 120-mer RNA probes with ≥ 95% identity to predicted NLR genes present in the *Triticeae* species barley (*Hordeum vulgare*), hexaploid bread wheat (*T. aestivum*), tetraploid pasta wheat (*T. durum*), red wild einkorn (*T. urartu*), domesticated einkorn (*T. monococcum*), and three goatgrass species (*Ae. tauschii*, *Ae. sharonensis*, and *Ae. speltoides*). For *Sr22*, six independent susceptible mutants were identified from 1,300 M_2_ families of mutated cultivar Schomburgk carrying the *Sr22*. The library was used for enriching of NLR wild-type sequences, as well as the six independent mutants. The NLR complements were sequenced using Illumina short-read sequencing and comparison of *Sr22* mutant reads to the de novo assembly of the wild-type NLR contigs identified 23 contigs that were mutated in two mutants, three contigs that were mutated in three mutants, and a single 3,408-bp contig that contained independent mutations in five of six mutants. This contig could be physically linked to an adjacent contig containing a polymorphism in the remaining sixth mutant. Thus, these six independent mutants were sufficient to directly identify a single gene corresponding to *Sr22*. The MutRenSeq was also successfully used in cloning of *Yr5*, *Yr7*, *YrSP* (Marchal et al. 2018), *Pm1a* (Hewitt et al. 2021a), *Sr26*, *Sr61* (Zhang et al. 2021), *Sr27* (Upadhyaya et al. 2021), *Lr13/Ne2*^*m*^ (Hewitt et al. 2021b), and *Sr9* (Zhang et al. 2023) genes (Supplementary Tab. S1). RenSeq allowed reducing the genome to a fraction of 14.5 Mb (8,235 contigs), which corresponds to only 0.09% of a hexaploid wheat genome. Capture arrays provide an effective way to reduce the complexity of large cereal genomes. Unfortunately, the gene of interest may be absent from capture arrays or in the case of MutRenSeq not all disease resistance genes are the NLR-like genes (e.g., *Lr34*, Krattinger et al. 2009).

Another strategy for reducing the wheat genome complexity is the utilization of previously mentioned chromosome flow sorting. Sánchez-Martín et al. (2016) used combination of EMS mutagenesis with chromosome flow sorting and Illumina short-read sequencing (MutChromSeq) to clone the powdery mildew resistance gene *Pm2a*. In MutChromSeq (Fig. 2), the chromosome carrying the gene of interest is flow sorted from each mutant and the respective wild-type parent and subjected to Illumina short-read sequencing. Wild-type reads are assembled into contigs, onto which mutant reads are mapped and inspected for mutation overlap. By applying the MutChromSeq pipeline to six susceptible mutants they identified 10-kb contig containing a full-length NLR-type gene, in which all mutants had the G/C-to-A/T transitions typical for the EMS. While initial linkage mapping is still necessary, laborious and time-consuming fine-mapping and chromosome walking are not required. The genes *Pm4a* (Sánchez-Martín et al. 2021), *Rht18* (Ford et al. 2018), *Lr14a* (Kolodziej et al. 2021), *Sr43* (Yu et al. 2023) as well as *Yr87/Lr85* (Sharma et al. 2024) have been also cloned by the MutChromSeq approach (Supplementary Tab. S1).

Although the chromosome flow sorting has been established for several plant species, its use may not be universal since it requires a significant (at least a 5%) difference in the chromosome size or GAA content (Giorgi et al. 2013). Such limitation may be bypassed by complexity reduction through RNA sequencing, which is a very efficient approach as only a small proportion of a genome harbors protein-coding genes. As mentioned above the wheat coding sequences represent only slightly over 1% of the genome. In 2022, Yu et al. (2022) proposed an approach termed MutRNASeq (Fig. 2), based on sequencing RNA of mutants, and used it to clone the *Sr62* resistance gene. *Sr62* was mapped to the 480 kb physical interval of *Ae. sharonensis* (AS_1644) chromosome 1S. RNA-seq of AS_1644 mapped on genome assembly of AS_1644 enabled to identify seven candidate genes in the mapped region. To identify *Sr62* among the candidate genes, they performed EMS mutagenesis of the Zahir-1644 introgression line carrying *Sr62*. RNA-seq reads of 14 mutants (> 91 million 150-bp paired-end RNA-seq reads per sample) were mapped to the assembled transcripts of the seven genes obtained by sequencing of full-length cDNA library of AS_1644 (98 million 150-bp paired-end reads). By that, they identified eight point mutations in the annotated *WTK5* gene among seven of the 14 mutants, which was subsequently verified to confer stem rust resistance. A comparable methodology was also used by Li et al. (2023a) to clone the *Lr47* resistance gene.

MutRNASeq was also utilized by Li et al. (2023b) to clone the powdery mildew resistance gene *Pm69*. The gene was mapped into the region with substantial structural variation, which caused suppression of recombination within the *Pm69* gene region. This prevented the use of published reference genome sequences as a skeleton for further high-density mapping. As an alternative approach, they attempted to use MutChromSeq and produced *pm69* loss-of-function mutants. However, they failed to flow-sorted chromosome 6B carrying the *Pm69* (only 47% purity), because it has similar size and GAA microsatellite content as 1B, 7B, 4B, and 5B chromosomes. The problems were solved using Oxford Nanopore Technology (ONT) long-read genome sequencing combined with MutRNAseq. ONT sequencing was used for whole-genome sequencing (23 × coverage) of the accession line. The candidate gene was identified by mapping RNA-seq reads from the wild type and four independent EMS-derived susceptible mutants onto contigs obtained by ONT whole-genome sequencing. Using this approach, named as ONT-MutRNA-seq, they identified eight expressed genes in the mapped region from which only one had five different point mutations in the four susceptible mutants.

Recently, two very similar methods called MutIsoSeq (Wang et al. 2023) and STAM (Sequencing trait-associated mutations, Ni et al. 2023) were developed (Fig. 2) to clone the wheat leaf rust disease resistance genes *Lr9/Lr58* and *YrNAM*, respectively. These methods utilize a combination of isoform sequencing (PacBio Iso-seq) of wild-type parental line and a deep RNA-seq of EMS mutants. The full-length cDNA, ranging up to 10 kb or more, produced by PacBio SMRT sequencing technology is used as a reference to which short-read RNAseq data of mutants are mapped. Transcripts which carry EMS-type mutations are identified and transcript mutated simultaneously in all mutant lines is a candidate to code the gene of interest. For *Lr9/Lr58*, seventeen independent susceptible mutants were identified out of 919 M_2_ families. Ten mutants together with the wild type were subjected to the MutIsoSeq pipeline, and only one transcript simultaneously mutated in all mutant lines was identified. The putative 3,504-bp *Lr9/Lr58* transcript sequence encoded protein with an N-terminal tandem kinase domain followed by a von Willebrand factor A (vWA) domain and a Vwaint domain in the C-terminus (*WTK6-vWA*). For *YrNAM*, the STAM pipeline was applied to the wild type and seven independent susceptible mutants. Only one transcript was identified to be mutated in six of seven susceptible mutants. The genomic sequence of *YrNAM* was obtained from whole-genome sequencing data of the accession line by identifying the contig matching the identified transcript. Comparing MutIsoSeq and STAM, MutIsoSeq retains multiple IsoSeq isoforms derived from the same gene, whereas STAM employs a non-redundant full-length transcriptome as a reference. MutIsoSeq and STAM do not require whole-genome/chromosome sequencing to identify a candidate gene as they only require an Iso-Seq dataset from the wild-type and RNA-seq data from multiple independent loss-of-function mutants that have a mutation in an exon. These approaches represent a low-cost option for rapid candidate gene identification. Nonetheless, the outcome is transcript sequence, and the genomic sequence of the gene must be generated.

## GWAS—an alternative to QTL mapping

Recently, the genome-wide association studies (GWAS) have emerged as an efficient alternative to the standard bi-parental QTL mapping. It enables the detection of QTLs without preparing a bi-parental population by exploiting historic recombination events accumulated in natural populations (Browning and Browning 2007). The selected natural population, consisting of hundreds of individuals, is high-throughput genotyped, and markers are ordered according to their position in a reference genome sequence. The reference sequence used may be a limitation, if it does not include the locus with the gene of interest. Similarly, only QTLs which are commonly present in the studied population can be identified. The association analysis between the ordered SNPs and phenotype results in identifying the genomic position(s) of markers or LD blocks associated with the tested trait variability (QTL loci). Such loci provide an ideal basis for gene cloning.

Arora et al. (2019) in the AgRenSeq approach used a combination of k-mer-based association mapping with RenSeq (resistance gene enrichment sequencing) to discover disease resistance genes from wild crop relative *Ae. tauschii*. They used a panel comprising 151 accessions and identified four non-redundant, high-confidence candidate stem rust resistance genes. Two of them corresponded to *Sr33* and *Sr45* resistance genes already cloned by map-based cloning and MutRenSeq, respectively (Periyannan et al. 2013; Steuernagel et al. 2016). Two other candidate *Sr* genes coincided with previously identified disease resistance genes *SrTA1662* (Olson et al. 2013) and *Sr46* (Yu et al. 2015). Only *Sr46* was functionally validated and therefore confirmed.

Later, Gaurav et al. (2022) applied a modified k-mer-based association mapping pipeline developed by Arora et al. (2019) on a sequenced panel of 242 *Ae. tauschii* accessions and successfully identified and cloned the stem rust and powdery mildew resistance genes *SrTA1662* and *WTK4*, respectively. The *SrTA1662* was associated with a 50-kb LD block which contained two genes, of which one encoded the NLR-type gene previously also identified by Olson et al. (2013) and Arora et al. (2019). The *WTK4* was mapped to a 320-kb LD block, which contained 19 genes, of which only *WTK4* represented a gene class previously reported to confer resistance to different pathogens. Both candidates were successfully verified by functional validation.

## Functional validation of candidate gene(s)

Functional validation of candidate gene(s) is the final step in the gene cloning process, and it serves to confirm that the candidate gene is responsible for the trait of interest. Candidate genes can be validated by methods of forward or reverse genetics. Validation by forward genetics includes mutagenesis followed by screening for loss-of-function mutants or indirect evidence can be provided by association mapping performed on large number of accessions differing in studied phenotype.

Reverse genetics requires development of a recombinant DNA construct specifically designed to express, down-regulate, or knock-out the target gene. The candidate gene or its fragment is cloned into an expression vector. The expression vector is subsequently delivered into the plant cell, usually by biolistic particle bombardment or agroinfiltration using *Agrobacterium tumefaciens*. The agroinfiltration can be hampered by the recalcitrance of many plant species/varieties to Agrobacterium infection, mainly due to the induction of plant defense responses. This has been recently improved by engineering an Agrobacterium strain expressing the type III secretion system and the AvrPto effectors from *Pseudomonas syringae* to suppress host defense responses. The modified Agrobacterium improved transformation efficiency of wheat, alfalfa, and switchgrass by 250–400% (Raman et al. 2022). In wheat, both stable and transient transformations have been successfully established and applied for candidate gene validation. Stable gene expression, down-regulation, or knock-out requires development of transgenic lines. In wheat, immature embryos of a cultivar are transformed and regenerated to adult plant. Stable transformation is often limited by a low plant regeneration efficiency resulting in few transformable genotypes. Recent studies have reported improvements in the efficiency of plant regeneration from tissue culture by overexpression of plant developmental regulator genes (Lowe et al. 2016). In wheat, co-delivery and overexpressing of GROWTH-REGULATING FACTOR 4 (CRF4)-CRF INTERACTING FACTOR 1 (GIF1) substantially improves transformed cell regeneration and increases the number of transformable cultivars (Debernardi et al. 2020). Unlike stable transformation, transient transformation is carried out on seedlings or leaf tissue and does not result in the permanent integration of foreign genetic material into the host genome, allowing for short-term gene expression (Schweizer et al. 1999). A major advantage of transient transformation is that it avoids the time-consuming, laborious generation, and maintenance of stable transgenic lines and could be used where there is a need to pre-screen many candidate genes. Additionally, ectopic expression in model organisms remains an alternative for functional validation.

Traditionally used reverse genetics approaches for candidate gene validation in wheat include transient gene expression (e.g., Srichumpa et al. 2005; Hurni et al. 2013; Singh et al. 2018), transformation of candidate gene into the background with contrasting phenotype (e.g., Mago et al. 2015; Chen et al. 2018; Wang et al. 2020), stable or transient down-regulation by RNA interference (RNAi; e.g., Yan et al. 2004; Rawat et al. 2016; Huai et al. 2022), and transient down-regulation by Virus-Induced Gene Silencing (VIGS; e.g., Zou et al. 2018; Sánchez-Martín et al. 2021; Pan et al. 2024). Apart from these traditional methods, the availability of genome editing approaches, including Transcription Activator-Like Effector Nuclease (TALEN; Hockemeyer et al. 2011) and Clustered Regularly Interspaced Short Palindromic Repeats (CRISPR)/Cas9 (Doudna and Charpentier 2014) technologies, offers a new avenue for candidate gene validation. TALEN and CRISPR/Cas9-mediated genome editing has been successfully applied in wheat to induce small deletions leading to subsequent disruption of a gene function. For example, TALEN was used to simultaneously target the three homoeologous copies of the wheat *MLO* gene (Wang et al. 2014). However, this technology is gradually being replaced by CRISPR/Cas9-mediated gene editing because of its simpler design, smaller construct size, and ability to target multiple sites or genes simultaneously. It also has the potential for complex applications such as base editing, sequence/gene insertion or deletion. Detailed protocols for some of these approaches are available online (www.wheat-training.com) or in published studies (e.g., Lee et al. 2015; Hayta et al. 2019).

## Conclusion

**Fig. 3:**
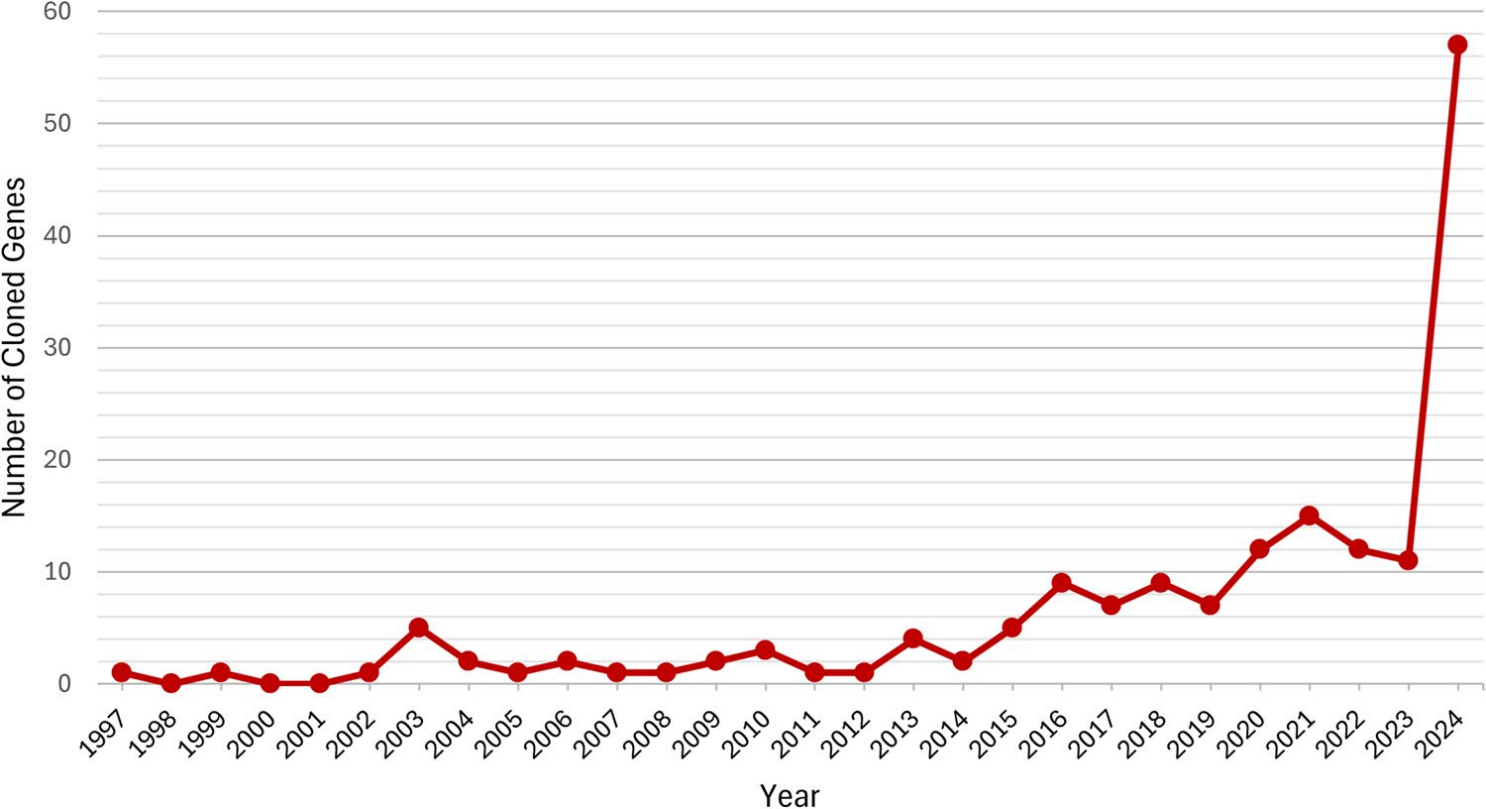
Dynamics of wheat gene cloning from 1997 to 2024. Note the significant increase in the number of successfully cloned wheat genes since 2014, driven by the publication of the draft bread wheat genome sequence and advances in the NGS sequencing and gene function validation approaches of reverse genomics

Over the past two decades, advancements in molecular techniques and genomics have significantly transformed gene cloning in wheat. The availability of the wheat genome sequence, combined with recent progress in next-generation sequencing technologies, has greatly facilitated map-based cloning and paved the way for new approaches, particularly those based on mutagenesis. As a result, the number of successful gene cloning projects has increased dramatically, with 172 genes now successfully cloned (Fig. 3). Of these, 68 genes were cloned using map-based cloning, 55 via homology-based methods, 21 through mutagenesis, 4 by GWAS-based approaches, and the remaining 24 using a combination of techniques (Supplementary Tab. S1, S2). Notably, a strong and reliable phenotype is a key advantage, as more than half of the cloned genes are associated with disease resistance (Supplementary Tab. S1).

Each cloning approach has its own set of advantages and limitations, and no single method is universally applicable. Homology-based cloning bypasses the labor-intensive candidate gene identification process, as it only requires functional validation. This method is particularly useful for cloning genes or alleles with multiple orthologs or paralogs, but it is limited to genes that have been previously characterized in other species and have corresponding orthologs in wheat. Map-based cloning is effective for cloning any single-copy gene, including major QTLs that can be reliably mendelized. However, this approach is challenging for genes located in regions with suppressed recombination. Mutagenesis-based approaches offer a direct path to candidate gene identification, bypassing the need for genetic mapping and reducing the time needed for cloning. These methods are especially suited for major genes with easily distinguishable phenotypes, such as disease resistance genes (Supplementary Tab. S1). GWAS has emerged as a promising tool for rapid candidate gene identification. However, it requires well-structured populations to minimize false positives and a sufficiently large population to provide high-resolution mapping with small LD blocks. Since GWAS typically detects common variants with moderate to high allele frequencies, rare variants may be missed, making it less suitable for cloning rare genes.

In summary, a range of approaches is now available to overcome the challenges posed by wheat’s large, complex, and highly repetitive genome. Gene cloning in wheat is becoming increasingly efficient, and the number of cloned genes will undoubtedly continue to grow, contributing to advancements in wheat improvement.

## Supplementary Information

Below is the link to the electronic supplementary material.

Supplementary file1 (XLSX 84 KB)
